# A Nuclear DNA Perspective on Delineating Evolutionarily Significant Lineages in Polyploids: The Case of the Endangered Shortnose Sturgeon (*Acipenser brevirostrum*)

**DOI:** 10.1371/journal.pone.0102784

**Published:** 2014-08-28

**Authors:** Tim L. King, Anne P. Henderson, Boyd E. Kynard, Micah C. Kieffer, Douglas L. Peterson, Aaron W. Aunins, Bonnie L. Brown

**Affiliations:** 1 U.S. Geological Survey (USGS), Leetown Science Center, Aquatic Ecology Branch, Kearneysville, West Virginia, United States of America; 2 USGS, Leetown Science Center, Fish Health Branch, Kearneysville, West Virginia, United States of America; 3 University of Massachusetts, Department of Environmental Conservation, Amherst, Massachusetts, United States of America; 4 USGS, Leetown Science Center, S. O. Conte Anadromous Fish Research Laboratory, Turners Falls, Massachusetts, United States of America; 5 University of Georgia, Warnell School of Forest Resources, Athens, Georgia, United States of America; 6 Cherokee Nation Technology Solutions, Leetown Science Center, Kearneysville, West Virginia, United States of America; 7 Virginia Commonwealth University, Department of Biology, Richmond, Virginia, United States of America; Institute of Biochemistry and Biology, Germany

## Abstract

The shortnose sturgeon, *Acipenser brevirostrum*, oft considered a phylogenetic relic, is listed as an “endangered species threatened with extinction” in the US and “Vulnerable” on the IUCN Red List. Effective conservation of *A. brevirostrum* depends on understanding its diversity and evolutionary processes, yet challenges associated with the polyploid nature of its nuclear genome have heretofore limited population genetic analysis to maternally inherited haploid characters. We developed a suite of polysomic microsatellite DNA markers and characterized a sample of 561 shortnose sturgeon collected from major extant populations along the North American Atlantic coast. The 181 alleles observed at 11 loci were scored as binary loci and the data were subjected to multivariate ordination, Bayesian clustering, hierarchical partitioning of variance, and among-population distance metric tests. The methods uncovered moderately high levels of gene diversity suggesting population structuring across and within three metapopulations (Northeast, Mid-Atlantic, and Southeast) that encompass seven demographically discrete and evolutionarily distinct lineages. The predicted groups are consistent with previously described behavioral patterns, especially dispersal and migration, supporting the interpretation that *A. brevirostrum* exhibit adaptive differences based on watershed. Combined with results of prior genetic (mitochondrial DNA) and behavioral studies, the current work suggests that dispersal is an important factor in maintaining genetic diversity in *A. brevirostrum* and that the basic unit for conservation management is arguably the local population.

## Introduction

Sturgeons (Acipenseridae) are one of two living groups of chondrostean fishes; the other group being the paddlefishes (Polydontidae). The fossil record suggests these were dominant fishes of the Permian period (∼200 Myr [1]) and owing to morphological similarities to their extinct relatives, modern sturgeons often are described as being evolutionarily static [2], [3]. Acipenserids also are notable for their anadromous and amphidromous life histories, unique benthic and life-history specializations, and the propensity for inter-genus and inter-species hybridization, the latter resulting in various levels of polyploidy which is slightly at odds with the fact that estimated mutation rates within the mitochondrial and nuclear genomes of acipenserids are reduced compared to other fishes [4], [5], [6]. The continued existence of these relic fishes is in jeopardy throughout North America, Europe, and Asia where nearly all sturgeon species have experienced overfishing, habitat degradation or loss, and obstruction of spawning areas. Much effort has been directed at understanding ecological factors associated with sturgeon biology [7] and behavior [8], [9] to address the prevailing conservation biology tenet that management planning must be framed in terms of providing conditions that will facilitate potential adaptation. For resource managers to plan for an evolutionary future for such “trust species,” they must have the means to identify evolutionarily distinct and significant lineages (e.g., species, metapopulations, populations, distinct population segments). However, some of the same characters that herald scientific interest (e.g., polyploidization, lengthy lifespans, long times to maturation, and intermittent semi-annual spawning) exacerbate efforts to identify and protect evolutionarily distinct lineages within each species.

Documenting heritable genetic information is a hallmark of contemporary conservation strategies for delineating management units and has previously been applied to sturgeons where recent molecular systematics studies have called into question the taxonomic foundations of the Acipenseriform classification, which has been historically based on morphological characters [10], [11], [12], [13]. Different gene regions, including some physically linked within the mitochondrial (mt) DNA molecule, have yielded differing phylogenetic interpretations of the Acipenseridae (see [13] for review). Efforts to resolve the molecular systematics of the Acipenseridae using nuclear (n) DNA sequences have either focused on a single gene region (18S rRNA, [5]) or on interspecific comparisons of repetitive DNAs observed as a result of genomic DNA digestion (*Hin*dIII [14], *Pst*I [6]). The former was complicated by the polyploid genome and provided no additional phylogenetic resolution, whereas the latter studies concur to some degree with molecular phylogeny observed with mtDNA sequences [6]. Unfortunately, not all sturgeon species have detectable levels of some satellite DNAs [14] and furthermore, the level of intraspecific variability has been shown to be greater than the interspecific divergence among species belonging to the same phylogenetic clade [6]. Given such equivocal results, some revision of Acipenseriform classification is needed to guide conservation efforts. Ultimately, this lack of genomic resources for sturgeons hinders mechanistic study (but see [15]).

Gene duplication and subsequent functional divergence is a fundamental process of adaptive evolution [16] and is particularly relevant in the Acipenseridae where the presence of evolutionary polyploidy ranging in a series from 4n-8n-12n times the ancestral haploid number [17] presents significant challenges for investigating the evolutionary processes shaping the nuclear genomes [4], [18], [19]. It is unclear whether these polyploid events resulted from complete genome duplications (autopolyploid), hybridization between species of different ploidy levels combined with genome doubling (allopolyploidy), or a combination of these processes [20]. Following the polyploid events that gave rise to extant sturgeon species, the random and gradual diploidization process [21] is assumed to have resulted in functional diploidy [22]; however, the degree to which their various polyploid nuclear genomes exhibit disomic inheritance is unknown.

The shortnose sturgeon *Acipenser brevirostrum* is an amphidromous species endemic to the large coastal rivers of eastern North America. This species is distinguished among all the living acipenserids by exhibiting the largest number of chromosomes, 372 [18]. *A. brevirostrum* was listed as an “endangered species” under the US Endangered Species Preservation Act of 1967 and remains so despite re-assessment in response to a 1994 petition to de-list populations in tributaries to the Gulf of Maine. Of significant note is that of 19 putative population units identified based on the species' perceived strong fidelity to natal rivers [23], [24] some river populations continue to exist, although much reduced, but in other rivers, the species has been extirpated. In most instances, spawning status is either unknown or indicated to be of limited extent [25], [23] further complicating the prediction of biological units that could respond to conservation measures.

To date, all published information on phylogeographic- and population-level structuring in *A. brevirostrum* has been assessed through nucleotide sequence variation detected in the maternally-inherited mtDNA. This is presumably due to the difficult nature of interpreting allelic data from the functionally polyploid (putatively hexaploid) nuclear genome [26]. The mtDNA research has primarily been focused on a moderately polymorphic 440 base pair segment of the control region (CR) adjacent to the tRNA proline gene. These findings are well documented in the peer-reviewed literature [27], [28], [29], [26], [30], [31] and are consistent both among studies and between researchers. Although results reflect a shallow gene genealogy (gene tree) for the *A. brevirostrum* mtDNA CR, analyses of haplotype frequencies at the level of putative individual populations showed significant differences among nearly all river/estuarine systems in which reproduction is known to occur. One prior study [31] concluded that although higher level genetic relationships exist (e.g., Northeast vs. Mid-Atlantic; Northeast vs. Southeast; Mid-Atlantic vs. Southeast; and other Mid-Atlantic regional subdivisions), *A. brevirostrum* appear to function in discrete populations, and that relatively low female-mediated gene flow exists between the majority of populations. This implies that effective dispersal among drainages within regions has been sufficient to prevent deep divergence within this species over evolutionary time scales.

*Acipenser brevirostrum* has been shown to possess the highest number of chromosomes (N = 362–372) among all the Acipenseriformes karyotyped to date [18]. These authors, however, could not determine the species' exact level of polysomy (hexaploid or dodecaploid). Contemporary cytogenetic techniques (including signals from fluorescent *in situ* hybridization) suggest *A. brevirostrum* is a hexaploid species [19]. While immensely complex, nuclear DNA-based approaches to *A. brevirostrum* conservation could identify significant levels of informative genetic variation because certain duplicated loci and repetitive DNA may lack functional constraints, thus allowing rapid accumulation of differentiation in DNA sequences [32]. Moreover, if the observed patterns of nuclear DNA diversity and variation differed from those empirically determined for the maternally-inherited mtDNA, this would inform biologists of the degrees of site philopatry or sex-biased dispersal for *A. brevirostrum*. However, no phylogeographic or population informative nuclear markers have been identified for *A. brevirostrum* [33].

To address this important research need and for the first time, allow an extensive assessment of the phylogeographic structure of *A. brevirostrum* from a multilocus nuclear DNA perspective, we characterized the inheritance of polysomic microsatellite DNA loci in shortnose sturgeon collected throughout the species' range using loci derived specifically from this species [34]. Because of the complex modes of inheritance underlying the putatively hexaploid genome, we scored each allele (fragment) as a dominant marker with two states, presence or absence, resulting in the production of a binary character matrix. We report on the findings of an extensive statistical comparison of the patterns in allelic variation to identify and assess the reproductive status of populations and to delineate functional units of management to aid in recovery planning.

## Methods

### Sample collection

**Figure 1:**
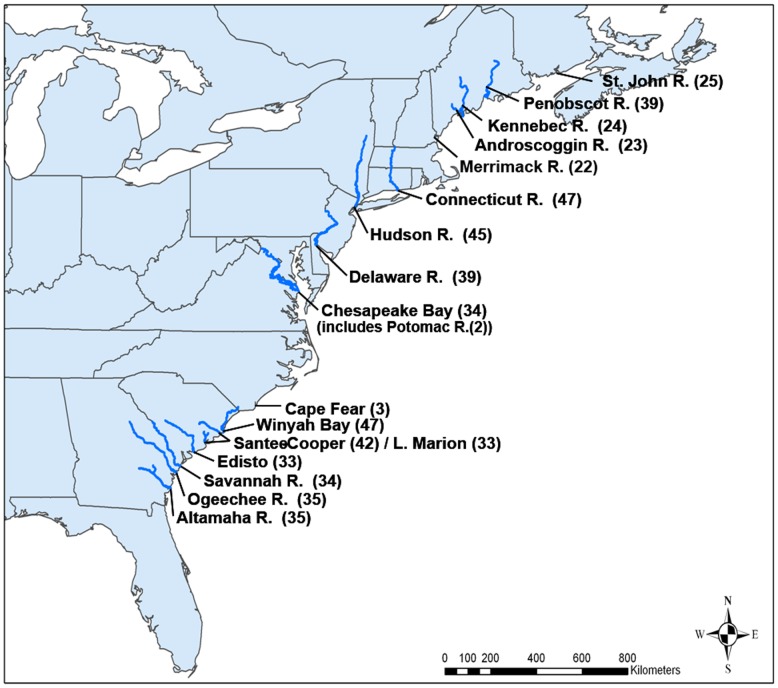
Map of a portion of the North American Atlantic Coast depicting the general location and sample size of 17 river and estuary collections of shortnose sturgeon (*Acipenser brevirostrum*) surveyed at 11 polysomic microsatellite DNA loci. Sample sizes are in parentheses.

While no tissue sampling was collected as part of this study, *Acipenser brevirostrum* (*n* = 561) were sampled from 17 river and estuarine systems representing the species' range (Figure 1) by researchers approved and permitted by the Institutional Animal Care and Use Committee of the U.S. Department of Commerce, National Oceanic and Atmospheric Administration, National Marine Fisheries Service. Sampling followed the guidelines mandated under NOAA Technical Memorandum NMFS-OPR-18 or NOAA Technical Memorandum NMFS-OPR-45. The mandated non-invasive procedures were that tissue (1.0 cm^2^ fin-clip) taken from soft pelvic fin was stored in 95% absolute ethanol or SDS/urea.

The ambiguous reproductive status of *A. brevirostrum* in the Potomac and Merrimack Rivers affected categorization of specimens. We chose to treat the Potomac River collection (*n* = 2) both as of unknown origin and as part of the large Chesapeake Bay-proper collection. The Merrimack River sample consisted of males collected at the same location and time; however, because eggs (*n* = 4) and embryos (*n* = 2) were collected in Spring 2009, we considered the Merrimack River collection as a reproducing population. Genomic DNA from ethanol preserved samples was extracted with the Gentra Puregene DNA kit (Qiagen, Valencia, CA) following the manufacturers guidelines for whole non-mammalian blood and resuspended in TE (10 mM Tris-HCl, pH 8.0, 1 mM EDTA). Previously extracted genomic DNA from other researchers was extracted using the methods described in [28]. DNA concentrations were determined by fluorescence assay [35], and the integrity of the DNA was visually inspected on 1% agarose gels [36]. SDS-urea preserved samples were also processed using these procedures with the exception that cell lysis was not necessary and samples were subjected directly to protein precipitation and alcohol purification. All DNA samples were quantified as described above and diluted to 100 ng/µl for use in PCR amplification.

A suite of 11 microsatellite loci previously identified from *A. brevirostrum* [34] was surveyed: *Abr*B438, *Abr*D10, *Abr*D114, *Abr*D135, *Abr*D141, *Abr*D193, *Abr*D236, *Abr*D332, *Abr*D345, *Abr*D379, and *Abr*D557. Optimized PCR mixes consisted of the following: 100–200 ng of genomic DNA, 1× PCR buffer (10 mM Tris-HCl, pH 8.3, 50 mM KCl), 2.0 mM MgCl_2_, 0.2 mM dNTPs, 0.4 mg/ml Bovine Serum Albumin, 2.0 U *Taq* DNA polymerase (Promega Corporation, Madison, WI, USA), 0.25 µM forward and 0.5 µM reverse primer, and 0.3 µM fluorescent labeled M13 primer in a total volume of 25 µl. Amplifications were carried out on either PTC-200 or PTC-225 Thermal Cyclers (Bio-Rad Laboratories, Hercules, CA) using the following cycling conditions: initial denaturing at 94°C for 15 min; 29 cycles of 94°C for 1 min; annealing temperature (56–66°C) for 45 sec, 72°C for 45 sec; 5 cycles of 94°C for 1 min; 53°C for 45 sec; 72°C for 45 sec; and final extension at 72°C for 10 min. Fluorescently labeled fragment analysis was performed using an ABI 3130XL Genetic Analyzer and binning of alleles was performed using GENESCAN 2.1 Analysis software and GENOTYPER 3.6 fragment analysis software (Applied Biosystems, Grand Island, NY). Each locus was independently scored, and each amplicon meeting the signal strength conditions specified (at least 10% relative to the strongest allele) and fitting into the appropriate size category (based on repeat motif and an assumed step-wise mutational pattern) was classified as an allele.

### Statistical analyses of polyploid microsatellite markers

For polyploid individuals, gene duplication, multiple alleles, and the mode of inheritance can lead to practical and statistical complications in allelic identification and interpreting summary and population-level statistics [37]. Genetic stock identification studies on other Acipenserids using polysomic (tetrasomic) markers [38], [39] estimated gene dosages by relative peak intensity from electropherograms. Because of the large number of alleles (fragments) observed in the putatively hexaploid *A. brevirostrum*, allele dosage could not be reliably estimated from GENESCAN runs preventing the application of standard population genetic diversity statistics that require genotype or allele frequencies for their calculation. Two prior groups [39], [40] provided a validated approach to this dilemma and as in those studies, we scored each allele (fragment) as its own psuedodominant locus with one of two states, presence or absence, resulting in the production of a binary character (or allele) matrix. When codominant markers are screened in higher order polyploid species, and scored as psuedodominant loci (i.e., as binary character state), it is not possible to estimate either allele frequencies or heterozygosities directly. Allele (loci) frequencies at 11 polysomic microsatellite DNA markers were estimated on the *A. brevirostrum* allelotype matrix using the method of [41] as implemented by GenAlEx 6.3 [42]. Only alleles with an overall frequency of >1% were used for these and other statistical analyses.

Allelotypes were analyzed for patterns of population ‘genetic’ structure and for regions of genetic discontinuity at both the individual and collection levels. Pair-wise genetic distances of each individual fish to all other individuals (simple match coefficient; [43]) were calculated using the binary distance routine in GenAlEx. When calculated across multiple loci for a given pair of samples, this is equivalent to the tally of state differences between the two DNA allelotype profiles. Principal coordinate analyses (PCO) were used to graphically compare the individual distances without imposing the appearance of a bifurcating evolutionary history (ordinated with PAlaeontological STatistics ver. 1.76, PAST, [44]).

A second individual-based analysis designed to infer population structure among collections and identify genetic discontinuity in the allelotype matrix was performed using the model-based clustering method of the program STRUCTURE 2.3.2 [45]. Due to complex migration patterns assumed to exist among disjunct populations, a sequential method of inferring clusters (*k*) was used by first identifying the “uppermost” hierarchical level of population structure followed by subsequent analysis of each cluster to identify within-cluster structure [46]. In the initial phase, *k* = 1 to *k* = 20 clusters were considered for the 17 collections using a burn-in of 20,000 followed by 50,000 iterations, and 20 independent runs for each *k*. The optimum number of clusters in the initial phase was identified using Δ*k* as described by Evanno et al. [46]. Subsequent analysis of each cluster tested *k* = 1 to *k* = C+3 (the number of collections (C) included in the subset plus three), with a burn-in of 20,000 followed by 50,000 iterations, and 20 runs for each *k*. In the within-cluster analyses, *k* also was determined using the Evanno et al. method [46].

Direct comparisons were made of two pair-wise population-scale distance measures, Jaccard's and Φ_PT_, for assessing the underlying structure contained in the allelotype matrix. Jaccard's similarity coefficient [47] was calculated using PAST as it is one of the most commonly used and recommended [48] ecologically-based measures of similarity between populations. A non-parametric Analysis of Similarity (ANOSIM) [49] was performed on Jaccard's distance metric (1-Jaccard similarity) measured for 17 collections of *A. brevirostrum* using PAST. The test statistic, R (resulting from the distance values being converted to rank values) and its significance were computed by permutation of group membership, with 10,000 replicates. R values were presumed to be proportional to genetic distance. AMOVA Φ_PT_ calculated in GenAlEx using the binary data was considered as a coefficient of dissimilarity [43], [42]. Pair-wise Φ_PT_ values and significance levels of the variance components (H_0_ = no genetic difference among populations; Φ_PT_ = 0), based on 10,000 permutations, were measured for all collections. The relationship between the ecological-based (Jaccard's) distance and the population genetic distance (Φ_PT_) matrices for all 17 collections was statistically assessed with a matrix regression analysis [50] performed by the MXCOMP routine in NTSYS-PC 1.8 [51].

Because a small number of effective migrants can have a profound effect on a small population, as the effects of drift can be large in the absence of balancing gene exchange, estimation of the number of migrants rather than simply the rate [52] is an important conservation consideration. We estimated the effective migrants per generation using Φ_PT_ for the amount of genetic differentiation at these selectively neutral microsatellite loci according to S. Wright's classical relationship [53]. Although this approximation of gene flow has been shown to be quite robust under a range of demographic conditions [54], [55] the empirical validity of substituting the binary character analog of F_ST_, Φ_PT_, into this equation is, to our knowledge, untested.

The evolutionary history among the 17 collections (geographic populations) of *A. brevirostrum* was inferred by analysis of the pair-wise population Φ_PT_ matrix using two methods. The non-metric multidimensional scaling (NMDS, [55]) option of PAST was used for visualization of the non-parametric monotonic relationship among the dissimilarity matrix, the Euclidean distance between collections, and the location of each collection in low-dimensional space. In addition, the Neighbor-Joining method [57], a clustering procedure based on the minimum-evolution criterion for phylogeographic trees (the topology resulting in the minimum total branch length at each step of the algorithm) was performed with MEGA4, [58]. The associated pair-wise Φ_PT_ distance matrix was subjected to clustering with 5000 bootstrap replicates using the program PAST.

To compare the genetic variation observed for nDNA (this study) with mtDNA [31] genomes of *A. brevirostrum*, two comparisons were made to visualize patterns among the 14 collections common to both studies. Ordination of the nDNA Φ_PT_ and mtDNA Φ_ST_ matrices was independently performed using the MDS option of PAST. A separate analysis of the relationship between the Φ_ST_ and Φ_PT_ pair-wise distance matrices was statistically assessed with the Mantel matrix regression analysis [50] performed by the MXCOMP routine in NTSYS-PC 1.8 [51].

Maximum likelihood assignment tests were used to determine the likelihood of each individual's multilocus allelotype being found in the collection from which it was sampled using the program AFLPOP 1.1 [59]. AFLPOP makes no assumption of Hardy-Weinberg equilibrium and has been shown to be robust when applied to binary-coded polyploid allelotypes [40]. Allelotype patterns observed from other analyses were used to group collections into various management units and to test the appropriateness of these groupings using changes in assignment success. Constituencies of evolutionarily significant groupings of populations (e.g., regions, management units, distinct population segments) were ultimately investigated using hierarchical structuring of genetic variation measured (AMOVA) for numerous combinations of collections [60].

## Results

### Population structure of shortnose sturgeon

A total of 181 alleles were observed at the 11 loci resulting in a binary character matrix of 181 loci (columns) by 561 individuals (rows) (summarized in Table 1). The numbers of alleles with frequencies ≥1% observed at the 11 loci ranged from 55 (Cape Fear, *n* = 3) to 152 (Hudson, *n* = 45). Estimated heterozygosity (assuming Hardy-Weinberg equilibrium) ranged from 10.4% (Cape Fear) to 19.3% (Hudson). A lower estimate of heterozygosity was observed among southeastern populations compared to mid-Atlantic and northeastern populations.

**Table 1:** Microsatellite allele (a.k.a. pseudodominant locus) counts, percentage of loci (alleles) polymorphic, number of private alleles, and number of common alleles across all loci for shortnose sturgeon (*Acipenser brevirostrum*) populations across the North American range.

Population	Saint John	Penobscot	Androscoggin	Kennebec	Merrimack	Connecticut	Hudson	Delaware	Chesapeake Bay	Cape Fear	Winyah Bay	Santee-Cooper	Lake Marion	Edisto	Savannah	Ogeechee	Altamaha
Sample size (n)	25	39	23	24	22	47	45	39	34	3	47	42	33	33	34	35	36
1No. Alleles (loci)	118	131	126	130	105	121	152	134	127	55	119	111	95	112	113	112	107
Polymorphism (%)	65.2	72.4	69.1	70.7	58.0	66.9	84.0	74.0	70.2	22.7	65.2	60.8	51.9	61.9	61.9	60.8	59.1
2No. Private Alleles	1	0	1	0	0	1	1	0	0	0	0	0	0	0	0	0	0
3No. Common Alleles (< = 25%)	11	16	13	16	6	12	24	12	11	0	7	3	4	4	5	6	2

The analyses were conducted on the binary character matrix.

1 Number of different fragments.

2 Number of bands unique to a single population.

3 Number of common alleles with frequency ≤25%.

**Figure 2:**
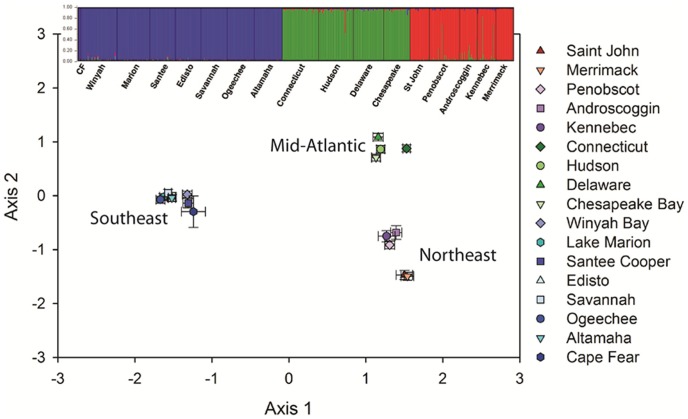
Combined graphical representation of principal coordinates (scatter plot) and STRUCTURE (histogram) analyses of 561 shortnose sturgeon (*Acipenser brevirostrum*) from 17 locations along the North American coast, surveyed at 11 polysomic microsatellite DNA loci. For the STRUCTURE histograms, each individual is represented by a single vertical bar, broken into *k* colored segments, the length of which is proportional to the membership fraction in each of the *k* clusters. Black lines partition the river samples.

At the individual level, the PCO scatter plot (Figure 2) indicated presence of three regional groups among the 17 surveyed river/estuarine systems. These regional groupings were: 1) *Northeast* - including five rivers from the Gulf of Maine (GOM), namely Saint John River, Canada; Penobscot, Kennebec, Androscoggin and Merrimack rivers; 2) *Mid-Atlantic* - including the Connecticut, Hudson, and Delaware rivers, and the Chesapeake Bay proper; and 3) *Southeast* – including the Cape Fear River, Winyah Bay, the Santee-Cooper, Edisto, Savannah, Ogeechee, Altamaha rivers, and Lake Marion. The number of inferred clusters (*k*) determined by STRUCTURE for the initial (uppermost hierarchical level) analysis was three, corresponding to the Northeast, Mid-Atlantic, and Southeast regional groupings identified by PCO (Figure 2). The PCO analysis clearly illustrated that the Southeastern cluster of individuals was the most divergent group of fish, separated from the other two regional groupings by a stronger zone of genetic discontinuity than the degree of separation between populations in the Northeast and Mid-Atlantic regions.

**Figure 3:**
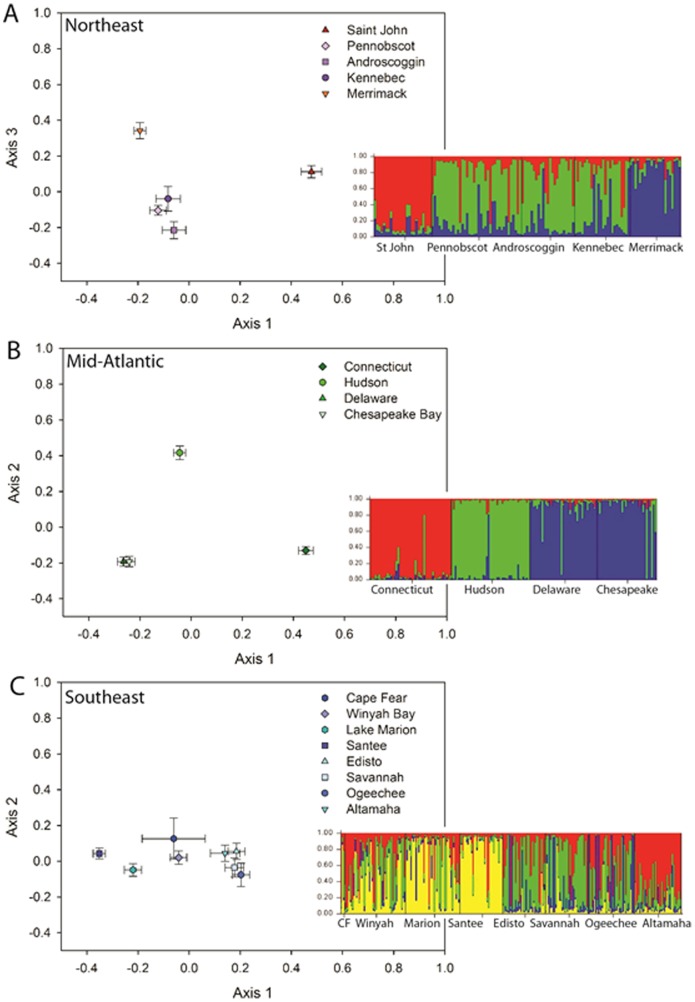
Combined graphical representation of sequential principal coordinates (scatter plots) and STRUCTURE (histograms) analyses of 561 shortnose sturgeon (*Acipenser brevirostrum*) surveyed at 11 polysomic microsatellite DNA loci. For the STRUCTURE histograms, each individual is represented by a single vertical bar, broken into *k* colored segments, the length of which is proportional to the membership fraction in each of the *k* clusters. Black lines partition the river samples. A) Northeast region collections; B) Mid-Atlantic region collections; C) Southeast region collections.

Due to the apparent complex migration patterns (zones of genetic discontinuity) existing among the three regions, a sequential method of inferring the number of clusters was employed to identify within-cluster structure (Figure 3; Evanno et al. 2005). Subsequent PCO analysis of the Northeast regional grouping of collections indicated a high degree of relatedness among the *A. brevirostrum* sampled from the Penobscot, Androscoggin, and Kennebec Rivers (Figure 3A), whereas fish sampled from the Saint John and Merrimack Rivers appeared well-differentiated from each other and from the other GOM rivers. The sequential STRUCTURE analysis of the Northeast cluster suggested panmixia among the *A. brevirostrum* sampled from the Penobscot, Androscoggin, and Kennebec Rivers and a moderate degree of differentiation between this group of collections and Saint John River to the north and Merrimack River to the south (Figure 3A). The level of differentiation of the Saint John and Merrimack Rivers from the other GOM collections did not appear as great as that seen among the mid-Atlantic collections or between the southeastern rivers and other collections (Figure 2).

PCO analysis of the Mid-Atlantic regional grouping indicated that *A. brevirostrum* sampled from Delaware River and Chesapeake Bay were genetically indistinguishable (mean and SE values were tightly clustered) and differentiated from Connecticut and Hudson River samples (Figure 3B). Likewise, the sequential STRUCTURE analysis also subdivided the mid-Atlantic cluster into three subclusters (Figure 3B). The two fish recently collected in Potomac River (during efforts to determine the existence of a reproducing population in this system) were genetically indistinguishable from other fish from Chesapeake Bay. This subsequent examination also revealed minimal overlap among the Connecticut River, Hudson River, and Delaware River/Chesapeake Bay collections; a pattern not readily discernible in the full analysis (Figure 2). Both PCO and sequential STRUCTURE analysis of *A. brevirostrum* from the Southeastern cluster of rivers suggested a low level of genetic differentiation among this group of populations, minimal structuring, and a moderate level of gene flow among collections throughout that region (Figure 3C).

At the population level, pair-wise Φ_PT_ values (Table 2, above diagonal) revealed that most (118/136; 87%) pair-wise comparisons among collections were statistically greater than zero (*P*<0.0004) indicating the presence of multiple populations displaying statistically significant differences in allele frequencies throughout the range of *A. brevirostrum*. Given the range and magnitude (0.040–0.249) of the Φ_PT_ values in question, the non-significant findings associated with this collection can likely be attributed to the inadequate sample size. The six remaining non-significant Φ_PT_ values were observed among geographically proximal collections within the Northeast and Southeast groupings (clusters). Pair-wise Φ_PT_ values were greatest among collections compared among the three major clusters and lowest among collections within these groupings. Low Φ_PT_ estimates were observed among collections in the Northeast (average 0.06) and Southeast (0.047) clusters. Moderately high Φ_PT_ estimates were observed among the four collections in the mid-Atlantic cluster (averaging 0.077) although the value between the Delaware River and Chesapeake Bay collections was 0.018. Considering the hierarchical AMOVA results (Table 3) in light of the individual-based PCO and STRCTURE analyses, seven distinct populations or groups of populations of *A. brevirostrum* were consistently supported: 1) St. John River in Canada, 2) three Maine rivers, 3) Merrimack River, 4) Connecticut River, 5) Hudson River, 6) Delaware River/Chesapeake Bay; and 7) southeast (all collections between the Cape Fear and Altamaha Rivers).

**Table 2:** Pair-wise Φ_PT_ among putative shortnose sturgeon populations (above diagonal) and estimates of the effective number of migrants per generation, *N_e_m* (below diagonal), for 17 collections of shortnose sturgeon (*Acipenser brevirostrum*) surveyed at 11 polysomic microsatellite loci.

Collection	Saint John	Penobscot	Androscoggin	Kennebec	Merrimack	Connecticut	Hudson	Delaware	Chesapeake Bay	Winyah Bay	Santee-Cooper	Lake Marion	Edisto	Savannah	Ogeechee	Altamaha
**Saint John**	-	0.068	0.077	0.068	0.100	0.191	0.162	0.175	0.155	0.253	0.289	0.269	0.269	0.278	0.280	0.277
**Penobscot**	3.43	-	***0.015***	***0.003***	0.065	0.116	0.094	0.107	0.095	0.189	0.219	0.207	0.189	0.201	0.205	0.203
**Androscoggin**	3.00	16.42	-	***0.013***	0.087	0.113	0.091	0.099	0.093	0.200	0.248	0.226	0.209	0.210	0.227	0.226
**Kennebec**	3.43	83.08	18.98	-	0.058	0.114	0.073	0.096	0.088	0.186	0.222	0.205	0.188	0.201	0.207	0.204
**Merrimack**	2.25	3.60	2.62	4.06	-	0.201	0.153	0.184	0.167	0.268	0.307	0.297	0.279	0.295	0.293	0.296
**Connecticut**	1.06	1.91	1.96	1.94	0.99	-	0.086	0.100	0.118	0.239	0.272	0.263	0.256	0.261	0.273	0.273
**Hudson**	1.29	2.41	2.50	3.17	1.38	2.66	-	0.067	0.075	0.179	0.217	0.208	0.188	0.196	0.210	0.201
**Delaware**	1.18	2.09	2.28	2.35	1.11	2.25	3.48	-	0.018	0.188	0.228	0.217	0.200	0.206	0.216	0.212
**Chesapeake Bay**	1.36	2.38	2.44	2.59	1.25	1.87	3.08	13.64	-	0.183	0.234	0.210	0.200	0.213	0.216	0.206
**Winyah Bay**	0.74	1.07	1.00	1.09	0.68	0.80	1.15	1.08	1.12	-	0.049	0.034	0.037	0.046	0.031	0.032
**Santee-Cooper**	0.62	0.89	0.76	0.88	0.56	0.67	0.90	0.85	0.82	4.85	-	0.044	0.043	0.043	0.046	0.069
**Lake Marion**	0.68	0.96	0.86	0.97	0.59	0.70	0.95	0.90	0.94	7.10	5.43	-	0.089	0.095	0.091	0.085
**Edisto**	0.68	1.07	0.95	1.08	0.65	0.73	1.08	1.00	1.00	6.51	5.56	2.56	-	***0.005***	***0.008***	0.020
**Savannah**	0.65	0.99	0.94	0.99	0.60	0.71	1.03	0.96	0.92	5.18	5.56	2.38	49.75	-	***0.00*** *7*	0.052
**Ogeechee**	0.64	0.97	0.85	0.96	0.60	0.67	0.94	0.91	0.91	7.81	5.18	2.50	31.00	35.46	-	0.020
**Altamaha**	0.65	0.98	0.86	0.98	0.59	0.67	0.99	0.93	0.96	7.56	3.37	2.69	12.25	4.56	12.25	-

Non-significant pair-wise Φ_PT_ probability values (H_0_ = No genetic difference among populations; Φ_PT_ = 0) based on 10,000 permutations values are in bold italics. Cape Fear River sample is not included due to inadequate sample size.

**Table 3:** Hierarchical AMOVA results for biogeographically relevant groups among 17 collections of shortnose sturgeon (*Acipenser brevirostrum*) surveyed at 11 polysomic microsatellite DNA markers.

	3-regions		5-regions		7-regions	
	Φ-statistic	% variance	Φ-statistic	% variance	Φ-statistic	% variance
Among regions (Φ_RT_)	0.158	16	0.164	16	0.170	17
Among populations within regions (Φ_PR_)	0.057	5	0.042	4	0.031	3
Within populations (Φ_PT_)	0.206	79	0.199	80	0.196	80

Non-parametric analysis of similarity (ANOSIM) of Jaccard's (ecological) distance resulted in a similar pattern of genetic differentiation in the pairwise R values (Table S1; below diagonal) and probability values (above diagonal) as observed with Φ_PT_ values. This correspondence was further reflected in the Mantel matrix regression analysis of the relationship between Φ_PT_ and Jaccard's distance measures, which indicated a strong, nearly absolute, correlation between the two distance metrics (*r* = 0.98, *P*<0.0001; Figure S1). As a result of the strength of this relationship, all subsequent distance-based analyses performed utilized the Φ_PT_ statistic because of its relationship to the standard diversity index used with codominant markers (F_ST_).

Multivariate (NMDS) analysis of the underlying structure contained in the population-level pair-wise Φ_PT_ matrix (data not shown) revealed a congruent pattern among the 17 collections with that observed with the individual-based PCO and in the STRUCTURE analysis (Figure 2) resulting in three major groupings of populations (Northeast, Mid-Atlantic, and Southeast) with varying degrees of clustering. The collections representing the southeastern populations and those from the GOM appeared tightly clustered (genetically similar). The Mid-Atlantic collections appeared to form three discrete groupings with the Delaware River and Chesapeake Bay collections the most closely related and more so than either was to the Hudson or Connecticut River collections. The Merrimack and Saint John River collections appeared moderately differentiated from the Maine collections.

**Figure 4:**
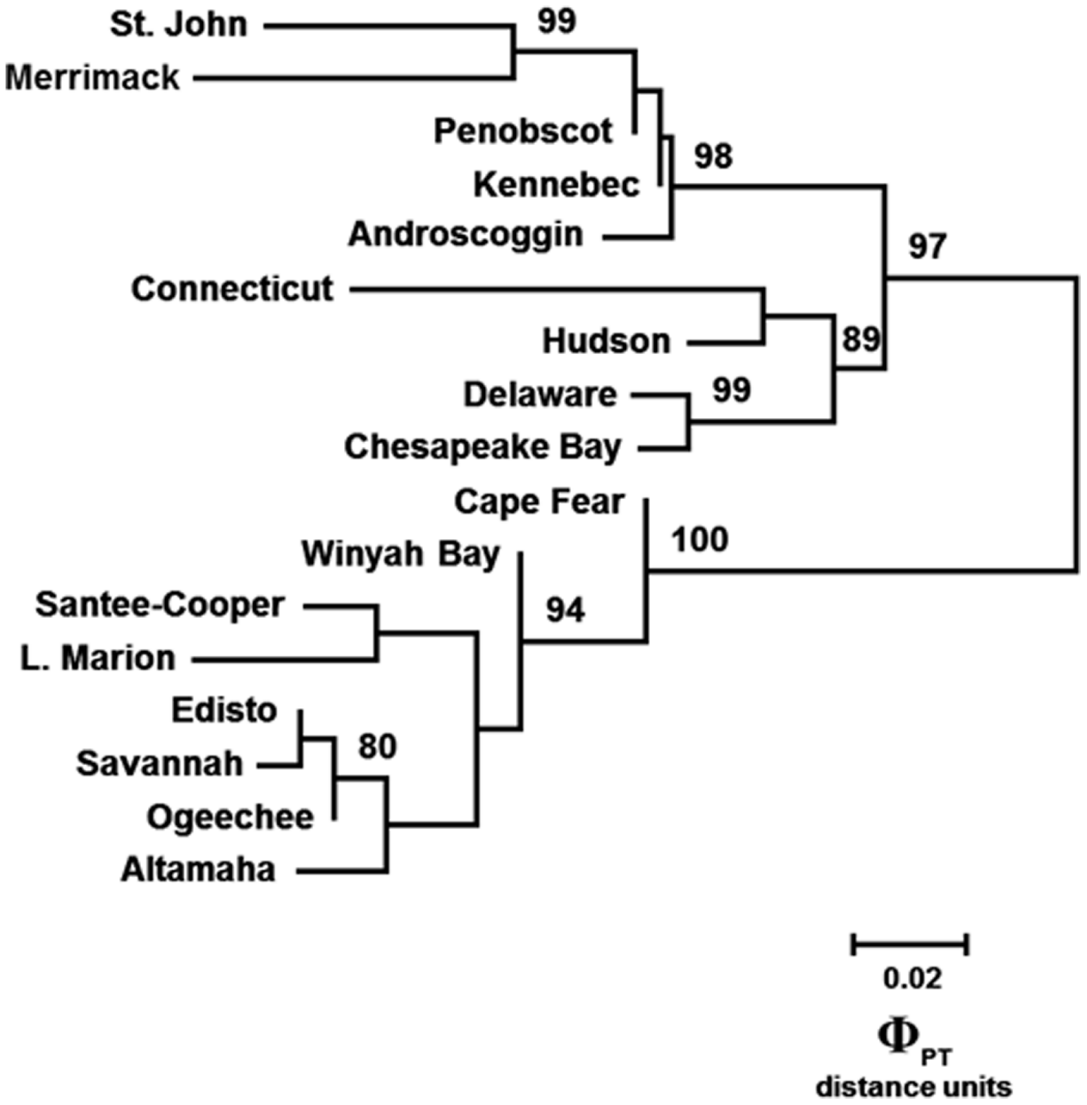
The evolutionary history among 17 collections of shortnose sturgeon (*Acipenser brevirostrum*) surveyed at 11 polysomic microsatellite DNA loci inferred from the pair-wise Φ_PT_ distance matrix using the Neighbor-Joining method [56]. Phylogenetic analyses were conducted in MEGA4 [57].

The underlying genetic structure of the Φ_PT_ matrix also was depicted with an unrooted neighbor-joining (N-J) tree (Figure 4), which illustrated high levels of differentiation among *A. brevirostrum* collections that mirrored those identified by the sequential PCO and STRUCTURE analyses. The deep level of differentiation (genetic discontinuity) among the three major groupings of collections was strongly supported in the backbone of the phenogram and the evolutionary distinctiveness of the populations or groups of populations was confirmed by high bootstrap support, in particular, the absolute (100%) bootstrap support distinguishing the southeast clade from all other collections, and the high bootstrap support for clades containing the Northeast (98%) and Mid-Atlantic (89%) collections. The high degree of genetic similarity observed with other analyses among the Penobscot, Androscoggin and Kennebec Rivers collections and between the Delaware River and Chesapeake Bay collections (99% bootstrap support) was confirmed with high bootstrap support for these pairings.

The effective number of *A. brevirostrum* migrants per generation, *N_e_m* (based on Φ_PT_), estimated between all pairs of collections (Table 2; below diagonal) was consistent with the patterns of differentiation observed with the individual- and population-based analyses (e.g., PCO, STRUCTURE, NMDS, and N-J). Clear zones of genetic discontinuity were evident as *N_e_m* among the three major regions was generally low, ranging from an average of 1.89 migrants between the Northeast and Mid-Atlantic collections to 0.89 between the Northeast and Southeast. The average *N_e_m* between the Mid-Atlantic and Southeast populations was 0.95. Estimates of *N_e_m* among collections within the three major regions were considerably higher ranging from 2.25–83.08 among the Northeast collections and 1.87–13.64 among the Mid-Atlantic collections. The range of *N_e_m* values among collections within the Southeast was 2.38–>100.

Quantitative estimates of hierarchical gene diversity among all collections also identified statistically significant genetic structuring; 16% (*P*<0.001) of the genetic variation occurred among collections (river/estuary populations) and 84% (*P*<0.001) was attributed to differentiation within collections. Of 11 hierarchical AMOVA analyses (Table S2), four models provided optimal delineation of genetic differentiation although the increase in variance among groupings over other models was minimal (1%). These four models all resulted in 17% (*P*<0.001) of the genetic variation occurring among groupings, 3% (*P*<0.001) occurring among populations within groupings, and 80% of the genetic variation was due to variation within collections. All four models, based on the subclusters identified by the PCO and STRUCTURE analyses, were variations either including or omitting the Saint John and Merrimack Rivers as distinct populations. All attempts to manipulate the southeast grouping of populations resulted in a decrease in variation among grouping components and an increase in the amount of within population variation.

The average correct assignment to collection of origin was 58.6% and ranged from 0% (Cape Fear River) to 97.8% (Connecticut River) (Table S3). With the exception of the Cape Fear River collection (*n* = 3), assignment to each collection was statistically greater than would be expected by chance (*P*<0.05). When the 17 rivers were pooled by the geographic regions identified in Figure 3, correct assignment to major grouping averaged 99.8% (Table 4). When pooled into two Northeast population groupings (i.e., Saint John separate from all other GOM collections), assignment to the GOM grouping was 100% (data not shown). A five-group model, (where the Northeast region included all GOM collections except the Saint John River) resulted in correct assignment to regional grouping in 99.1% of comparisons (Table S4).

**Table 4:** Assignment to three groupings of origin model consisting of 17 collections of shortnose sturgeon (*Acipenser brevirostrum*) surveyed at 11 polysomic microsatellite DNA markers.

allocated to	Northeast	Mid-Atlantic	Southeast
**Northeast**	132	0	0
**Mid-Atlantic**	1	165	0
**Southeast**	0	0	254
**Assignment %**	99.1	100	100

Mis-assigned individuals are distributed vertically.

### Comparison of the patterns nDNA and mtDNA

**Figure 5:**
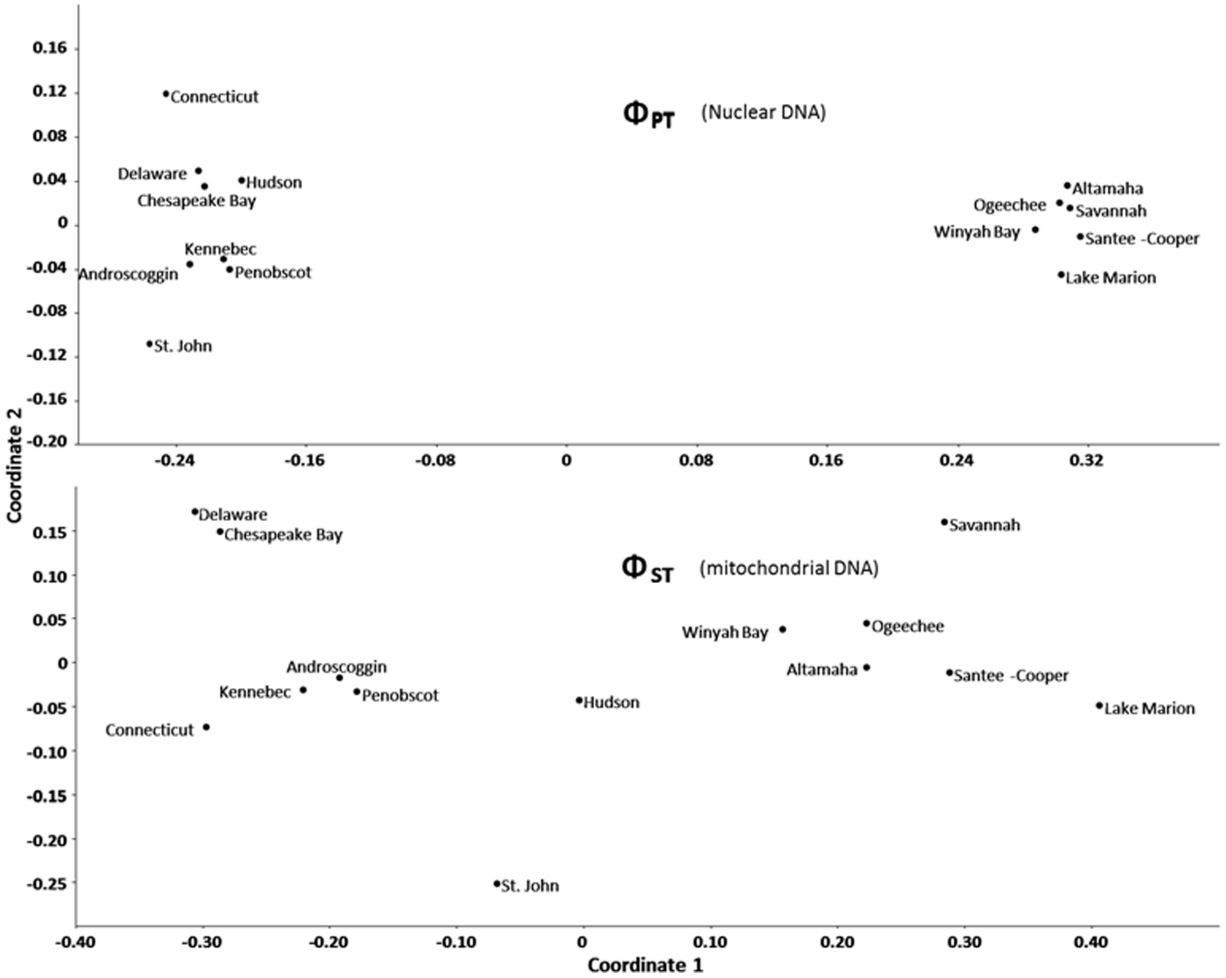
Results of independent multidimensional scaling analyses of pair-wise a) Φ_PT_ (nuclear DNA; Table 4, this study) and b) Φ_ST_ (mitochondrial DNA; Table 5 of Wirgin et al. [30]) matrices for 14 *Acipenser brevirostrum* collections that are in common between the two studies.

Mantel analysis comparing the pair-wise Φ_PT_ (this study) and Φ_ST_ (from Table 5 of Wirgin et al. [31]) distance matrices for 14 shared collections of Atlantic coast collections of *A. brevirostrum*, identified a strong statistical relationship (correlation coefficient *r* = 0.84, *P*<0.0001; Figure S2) between the variation detected in the two genomes. Furthermore, nDNA and mtDNA data yielded concurrent depictions of the presence of three major groupings representing the northeastern, mid-Atlantic, and southeastern populations (Figure 5). Moreover, similar levels and patterns of genome differentiation were observed among the mid-Atlantic groups: Connecticut River, Hudson River, and Delaware River/Chesapeake Bay. The respective scatter plots also suggest the presence of at least three regional metapopulations: Northeast (i.e., Penobscot, Kennebec, and Androscoggin Rivers), Mid-Atlantic (Delaware River and Chesapeake Bay), and Southeast (Altamaha, Winyah Bay, Santee-Cooper, Edisto, Savannah, Ogeechee, and Cape Fear Rivers, and Lake Marion).

## Discussion

### Phylogeography

#### Allelotypes

This study represents the first report of nuclear DNA variation in the higher-order polyploid *Acipenser brevirostrum*. The markers uncovered sufficient diversity that each of the 561 individuals surveyed possessed a unique multilocus allelotype. Alleles detected at these loci, scored as binary allelotypes, allowed robust qualitative and quantitative assessment of population structuring, similar to that conducted on disomically-inherited codominant markers. This study applied multivariate ordination and Bayesian clustering, assessed statistical significance (i.e., value>zero) of pair-wise distance metrics, performed matrix regression analyses, estimated the evolutionary history among populations via phylogenetic algorithm (phenogram), conducted hierarchical partitioning of variation (AMOVA), and assessed the success of likelihood assignment testing. Notably, the results of all statistical approaches used to assess phylogeographic structure resulted in consistent findings.

#### Identifying units of management

Owing to their morphological similarity to lower Jurassic (∼200 MYBP) Acipenseriformes [61], *Acipenser* species such as *A. brevirostrum* are considered to be evolutionarily static and are often referred to as phylogenetic relics. Intraspecific examination of the nuclear genome has revealed the presence of considerable allelic diversity and differentiation that appears to be reflective of the actions of various evolutionary processes. Phylogeographically, these findings suggest the presence of similar levels of genetic diversity and variation among the collections punctuated with a series of genetic discontinuities of varying “depth” across the species' range that could indicate demographic independence, regional adaptation, and reflect vicariant geographic events. Populations sampled within these regional groupings exhibited shallow but statistically significant differentiation, which were congruent with theoretical estimates of gene flow. Moreover, patterns of population relatedness were consistent with the observations of [23] that populations at both ends of the species' range are more dispersive than those in the middle. A possible explanation for elevated geneflow within the northern and southern collections could be the greater geographic proximity of rivers in these areas relative to those in the mid-Atlantic region leading to higher levels of straying.

Upon inspection of the patterns of allelotypic variation at the individual and population scales in the nDNA two major (“deep”) zones of genetic discontinuity are inferred from all analyses conducted 1) separating the Northeastern and Mid-Atlantic collections and 2) delineating the Mid-Atlantic and Southeastern populations. Moreover, narrower (“shallow”) zones of genetic discontinuity were evident among Saint John, Merrimack and the three Maine rivers in the Northeast region, and between the Connecticut and Hudson River collections and between Hudson River and an apparent Delaware River/Chesapeake Bay metapopulation (unconfirmed) within the mid-Atlantic region. This implies there are seven demographically and evolutionary distinct lineages across the range and that within the United States portion of the *A. brevirostrum* range, six lineages are relevant in conservation considerations. In addition to support for recognition of these zones of discontinuity in clustering, the phylogeographic analysis, AMOVAs, and assignment testing, all suggested there are low levels of theoretical gene exchange between collections on either side of these genetic discontinuities.

The presence of demographically distinct and evolutionary significant lineages delineated by zones of genetic discontinuity is consistent with the findings of researchers assessing behavioral patterns in *A. brevirostrum*. Parker [9] and Parker and Kynard (unpublished data) found that under common garden conditions, *A. brevirostrum* were locally adapted to particular rivers. These researchers demonstrated differences in the innate dispersal patterns in early life stages of *A. brevirostrum* from Connecticut River versus sturgeon of Savannah River origin, suggesting that *A. brevirostrum* are likely behaviorally adapted to unique features of their watershed. Parker and Kynard [62] also found that *A. brevirostrum* from different rivers can have different migration strategies. Similar adaptive differences have been inferred for other sturgeon species including *Acipenser fulvescens* (Wolf and Menominee rivers [Kynard and Parker unpublished data]), *A. transmontanus* (Sacramento and Kootenai rivers [63]), and *A. oxyrinchus oxyrinchus*/*A. o. desotoi* (Hudson and Suwannee rivers [24]).

#### Inter-genomic comparison

Comparison of nuclear genetic diversity observed in this study with that identified previously in mtDNA sequence variation ([31] and references therein) illustrates that the patterns of variation in the two *A. brevirostrum* genomes were qualitatively consistent. Similarly, the observation from the NMDS scatter plots in the two studies (Figure 5) that pair-wise Φ_ST_ values on average were approximately twice the pair-wise Φ_PT_ values for the same suite of collections is consistent with hyper-polymorphism associated with microsatellite evolution ([64], [65]) and the expectation of allele size homoplasy associated with polyploidy and an artifact of scoring alleles as binary types. Ultimately, this is the quantitative ‘penalty’ realized because allelotypic diversity is likely to be an underestimation of the actual differentiation that exists among populations; particularly for those that have experienced extended reproductive isolation. Although quantitative variation and molecular variation are correlated, adaptive population structuring often far exceeds neutral population structuring even for populations diverging over contemporary time ([66], [67]). Therefore, the estimates of allelic differentiation detected at neutral loci in this study are likely an underestimation of the actual divergence. Lastly, a component of the greater mtDNA haplotype differentiation relative to nuclear DNA differentiation could reflect gender-mediated gene flow between adjacent populations and resulting in reduced philopatry (i.e., sex-biased dispersal) of males throughout the range.

The phylogeographic and genomically-congruent patterns predicting genetic structuring of *A. brevirostrum* have profound management implications, foremost being the strong indication of regional structure. Specific cases within each region provide evidence that the fundamental units of management generally are the populations located in rivers and estuaries. However, because interpretations of the delineation of those distinct population segments differ depending upon the genome under investigation (7–9 based on mtDNA versus 5 if based on nDNA), we are faced with the need to specify evolutionarily relevant thresholds for deciding statistical significance of haplotype or allelotype frequencies. Ultimately, the approach that may offer maximum flexibility in reaching recovery goals may be to allocate (or nest) biological units that are most likely to respond to management within regions. The following two sections examine specific cases and the suggestion that a metapopulation approach may serve best to illustrate the structure of gene flow in this species.

### Cases in point

#### Saint John's River

Differentiation among the Northeast populations is on the whole less than observed for the Mid-Atlantic populations but greater than among the Southeast populations. Within the Northeast group, the Saint John River, Canada collection constitutes a population that is appreciably differentiated from the GOM collections at a number of levels. Foremost, the degree of gene differentiation between Saint John River and the GOM is “shallow”. This “shallowness” of may be attributed to the fact that these populations are relatively young (due to recent deglaciation of the region) as compared to more southerly distributed populations based on the observed levels of divergence. In addition, designation as a distinct management unit may be warranted because 1) the Saint John River *A. brevirostrum* population's age-to-reproduction is different than other GOM sturgeon, 2) it is the northernmost reproducing population, and 3) it experiences differences in control of exploitation, management of habitat, and conservation status.

#### Gulf of Maine

The GOM collections analyzed in this study included a recently obtained sampling from the Merrimack River. These *A. brevirostrum* exhibited patterns of nDNA variation that suggest the collection is genetically differentiated from the other GOM collections. Such an interpretation should be made with caution because the sample consisted of 22 males collected at the same location and time, and because the level of differentiation is not as great as that observed among other collections proposed as distinct evolutionary lineages. Choosing to consider this collection as a distinct evolutionary lineage would serve the precautionary principle and conserve biodiversity.

#### Potomac River

Recent captures of adult *A. brevirostrum* in the Potomac ([68], [69]) and Merrimack Rivers (this study) have raised questions concerning the status of this endangered species and available habitat in these river systems. Fishery managers require empirically supported prioritization schemes to protect existing diversity. It is important, therefore to determine if these observations are indicative of a discrete natal population or simply the result of migratory foraging behavior of fish from nearby populations. Analysis of mtDNA sequences from four Potomac River *A. brevirostrum* identified haplotypes also found in fish from the Chesapeake Bay and Delaware River [28]. This suggested the fish (or their mothers) were genetically related to other collections made in the Chesapeake Bay proper and Delaware River. Grunwald et al. 2002 suggested the fish might have been migrants from the Chesapeake and Delaware Canal [28]. Individual-based analyses of the two Potomac River *A. brevirostrum* sampled for the present study strongly suggested the fish are genetically part of a Delaware River/Chesapeake Bay metapopulation (Figure 3B). It will require significant additional resources to determine whether a reproducing population of *A. brevirostrum* exists within the Chesapeake Bay proper or whether the collections made there simply represent fish on foraging forays from the Delaware River population. Without recolonization and reestablishment of a reproducing population somewhere among the Chesapeake Bay rivers, a large gulf will continue to exist among northern and southern *A. brevirostrum* populations.

#### Savannah River supplemental stocking program

Early resource management efforts for *A. brevirostrum* included the release of hatchery-reared juveniles to supplement Savannah River populations [70]. Although conducted as an experimental program from 1984–1992, approximately 97,000 juveniles were introduced. This management effort has potentially altered the population genetic structure of the targeted river system as well as the southeast metapopulation. Smith et al. [70] estimated that 39% of the Savannah River's ‘breeding’ adults were of hatchery origin. It has been suggested that because *A. brevirostrum* were released as larger juveniles during this supplemental stocking program to allow marking, the fish were not imprinted to the natal stream and may have effectively strayed to adjacent river systems, i.e., traveled to non-natal rivers for reproduction, thereby having a homogenizing effect on impacted river populations [29]. However, it is unknown if effective introgression of hatchery-origin fish into the wild populations of any southeast river has occurred. No strong signal supporting or refuting a discernible impact on the southeast metapopulation by the Savannah River supplemental stocking is apparent from this study. Due to the geographic proximity of the Edisto and Ogeechee rivers to the Savannah River, the degree of evolutionary similarity among these rivers (e.g., in assignment testing) could be the result of natural straying or a homogenizing effect of the supplemental stocking program.

### Metapopulations

The current paradigm is that *A. brevirostrum* stray less often than the congeneric *A. oxyrinchus* [71]. However, at least two additional groupings involving adjacent river and estuarine systems have been identified in this study that exhibit strong signatures of functioning metapopulations (i.e., Maine rivers and Delaware River/Chesapeake Bay). Moreover, biologists tagging and tracking fish in Connecticut River recaptured two *A. brevirostrum* that originally were tagged and released in Hudson River [72]. Although this latter finding does not confirm effective movement (i.e., gene flow) between these river systems, evidence suggests some degree of straying and recolonization from adjacent southeastern rivers is possible. Indeed, if the strong signal of metapopulation structure observed among the southeastern rivers in this study resulted from the straying of stocked Savannah River *A. brevirostrum*, the effective population sizes of the affected rivers are indeed minuscule and the supplementation program must have been one of the most successful such efforts on record.

In addition to the demographically discrete and evolutionarily significant lineages identified for *A. brevirostrum* within the U.S., three metapopulations and other individual river populations delineated within each discrete lineage may be considered distinct management/recovery units for future recovery planning purposes. The three possible metapopulations are the: 1) Maine rivers (i.e., Penobscot, Kennebec, and Androscoggin rivers), 2) Delaware River and Chesapeake Bay proper, and 3) the Southeast assemblage (Cape Fear River, Winyah Bay, Santee-Cooper, Edisto, Savannah, Ogeechee, and Altamaha rivers, and Lake Marion). Population biology theory predicts that lower dispersal and associated gene flow leads to decreased genetic diversity in small isolated populations, which generates adverse consequences for fitness, and subsequently for demographic stability. Given recent tagging data suggesting *A. brevirostrum* migrate to adjacent rivers to a greater extent than previously believed [70], [71], [73], [74], [75], concomitant with the identification of at least three metapopulations within the species' range, this interpretation appears to bode well for the demographic fitness of some southern *A. brevirostrum* populations.

## Conservation Implications

This study presents evidence that sufficient levels of genetic diversity are present in the *A. brevirostrum* nuclear genome to discriminate evolutionarily significant lineages of management relevance. The variation detected was highly phylogeographically congruent with the inferences based on the mtDNA control region ([31] and references therein). Moreover, these nDNA analyses detected statistically significant differences in allelotype frequencies among most collections. Four regional zones of genetic discontinuity were inferred from the patterns of genetic variation across the range of *A. brevirostrum* (two shallow zones in the Northeast and two deep zones dividing three regions) that likely delineate seven demographically discrete and evolutionarily significant lineages, each with differing adaptive potential for this species. These zones of inferred genetic discontinuity represented deeper levels of differentiation and a higher degree of reproductive isolation than that typically attributed to population-level differentiation; groups of populations of evolutionary significance which may warrant distinct conservation considerations. Perhaps more notable than the delineation of evolutionarily significant lineages, was the identification of at least one putative metapopulation within each of the three major regional groupings of populations, a finding that is encouraging as migration may help stave localized extinctions. It should be noted that while an increased level of gene flow is present within these putative metapopulations, a demographic connection has yet to be established and documentation of such a link should be considered a high priority research need. Moreover, many of the populations within an evolutionarily significant lineage were genetically differentiated to some degree and each geographic population is subjected to differing threats [75]. Based on patterns observed from these multilocus allelotypes, the basic unit for management and conservation (for recovery planning) of *A. brevirostrum* is arguably the individual (local) population.

Assuming conservation of local populations within a metapopulation is the acceptable focus, restoration of effective connectivity among currently fragmented *A. brevirostrum* populations should be a prominent recovery goal; the allelotypic patterns observed in this study provide guidance by facilitating understanding of where and how such efforts could be attempted. For example, the best available information suggests that individual rivers and estuaries along the North Carolina coast do not currently support reproducing populations of *A. brevirostrum*. If the distance to North Carolina (or elsewhere) rivers that could support a reproducing population exceeds the vagility of sturgeon inhabiting the southeast or Delaware River/Chesapeake Bay metapopulations, targeted translocations or restorative supplementation may represent plausible restoration strategies. In contrast, rivers geographically south of the Altamaha River historically occupied by shortnose sturgeon (i.e., Satilla, St. Marys, and St. John Rivers), no longer support reproducing *A. brevirostrum* populations. The shoreline distances of these rivers to the Altamaha River are similar to that observed among the major rivers comprising the Southeast metapopulation. Given the recent findings of moderate physical migration [74] and our implications for effective migration (gene flow) among *A. brevirostrum* inhabiting many of the rivers in the Southeast metapopulation, it seems logical that if one or more of these southernmost rivers provided suitable spawning and rearing habitat, *A. brevirostrum* would have effectively colonized this region. Therefore, habitat characterization and/or restoration in these southernmost rivers could facilitate range expansion.

Gene diversity estimates for *A. brevirostrum* have been shown to be moderately high in both nuclear (this study) and mitochondrial ([29], [30], [31]) genomes. Although rates of genetic diversity loss in polyploids versus diploids (functional sensu) has not been characterized for sturgeon, the nDNA and mtDNA studies performed to date suggest that dispersal is a very important factor maintaining genetic diversity in shortnose sturgeon. The gene diversity estimates may be indicative of larger effective population sizes than previously assumed. However, even at a very local spatial scale in a metapopulation consisting of moderate-density populations interconnected by considerable dispersal rates, genetic diversity can erode and directly affect the fitness of individuals. Gene flow estimates do not capture the intra-specific variation in individual behavior related to vagility, which is strongly affected by habitat fragmentation and population/metapopulation history. From a biodiversity conservation perspective, future success in *A. brevirostrum* management could benefit from both in-depth demographic and genetic analyses. It should be considered a high-priority research need to better delineate population structure within the evolutionarily significant lineage framework suggested by these data.

## Supporting Information

Figure S1 **Scatter plot illustrating the significant correlation (** ***r*** **= 0.98;** ***P*** **<0.0001; Mantel analysis) between Jaccard and Φ_PT_ pair-wise distances for 17 collections of shortnose sturgeon (** ***Acipenser brevirostrum*** **) surveyed at 11 polysomic microsatellite DNA loci.**
(TIF)

Figure S2 **Scatter plot depicting the Mantel matrix regression analysis comparing the mtDNA Φ_ST_ matrix for 14 Atlantic coast collections of shortnose sturgeon (** ***Acipenser brevirostrum*** **) (Wirgin et al.** [30] **) and the nuclear DNA Φ_PT_ pair-wise distance matrix (this study) for the same collections surveyed at 11 polysomic microsatellite DNA loci.** The correlation coefficient (*r*) for this analysis was 0.84 (P<0.0001).
(TIF)

Table S1 **Pair-wise R values (below diagonal) and Bonferroni-corrected probability values (above diagonal) from the non-parametric Analysis of Similarity (ANOSIM) (Clark 1993) on Jaccard's distance metric (1-Jaccard similarity) measured among 17 collections of shortnose sturgeon (** ***Acipenser brevirostrum*** **) surveyed at 11 polysomic microsatellite loci.**
(DOC)

Table S2 **Hierarchical structuring of genetic variation was measured for numerous combinations of shortnose sturgeon (** ***Acipenser brevirostrum*** **) collections using analysis of molecular variance (AMOVA).** Significance levels of the variance components were based on 1000 permutations. Abbreviations are as follows: NE = Northeast regional grouping includes Saint John River (SJ), Canada, Penobscot, Kennebec, Androscoggin and Merrimack rivers; Mid-Atlantic regional grouping includes the Connecticut (CT), Hudson (H), and Delaware (DE) rivers, and the Chesapeake Bay proper (CB); and the 3) SE = Southeast regional grouping includes the Cape Fear River (CF), Winyah Bay (WB), Santee-Cooper (S-C), Edisto (E), Savannah (S), Ogeechee (O), and Altamaha (ALT) rivers, and Lake Marion (LM).
(DOC)

Table S3 **Assignment to collection of origin for 17 shortnose sturgeon (** ***Acipenser brevirostrum*** **) collections surveyed at 11 polysomic microsatellite DNA markers.** Mis-assigned individuals are distributed horizontally.
(DOC)

Table S4 **Assignment to proposed grouping (five groupings model) in shortnose sturgeon (** ***Acipenser brevirostrum*** **) surveyed at 11 polysomic microsatellite DNA markers.** The overall correct assignment rate to proposed grouping was 99.1% (522/527). Mis-assigned individuals are distributed vertically. Northeast regional grouping includes Saint John River (SJ), Canada, Penobscot, Kennebec, Androscoggin and Merrimack rivers; and the Southeast regional grouping includes the Cape Fear River (CF), Winyah Bay (WB), Santee-Cooper (S-C), Edisto (E), Savannah (S), Ogeechee (O), and Altamaha (ALT) rivers, and Lake Marion (LM).
(DOC)
