# Supplementary figures and images for: A Nuclear DNA Perspective on Delineating Evolutionarily Significant Lineages in Polyploids: The Case of the Endangered Shortnose Sturgeon (Acipenser brevirostrum)
Source: PLoS One. 2014 Aug 28;9(8):e102784. doi: 10.1371/journal.pone.0102784 (PMC4148239; doi:10.1371/journal.pone.0102784)

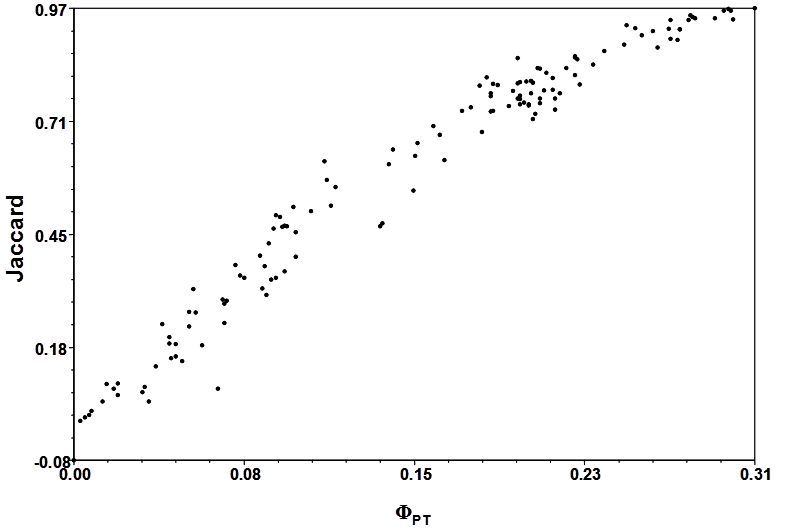

Supplement: Figure S1 — Scatter plot illustrating the significant correlation ( r = 0.98; P <0.0001; Mantel analysis) between Jaccard and ΦPT pair-wise distances for 17 collections of shortnose sturgeon ( Acipenser brevirostrum ) surveyed at 11 polysomic microsatellite DNA loci. (TIF) [file pone.0102784.s001.tif]

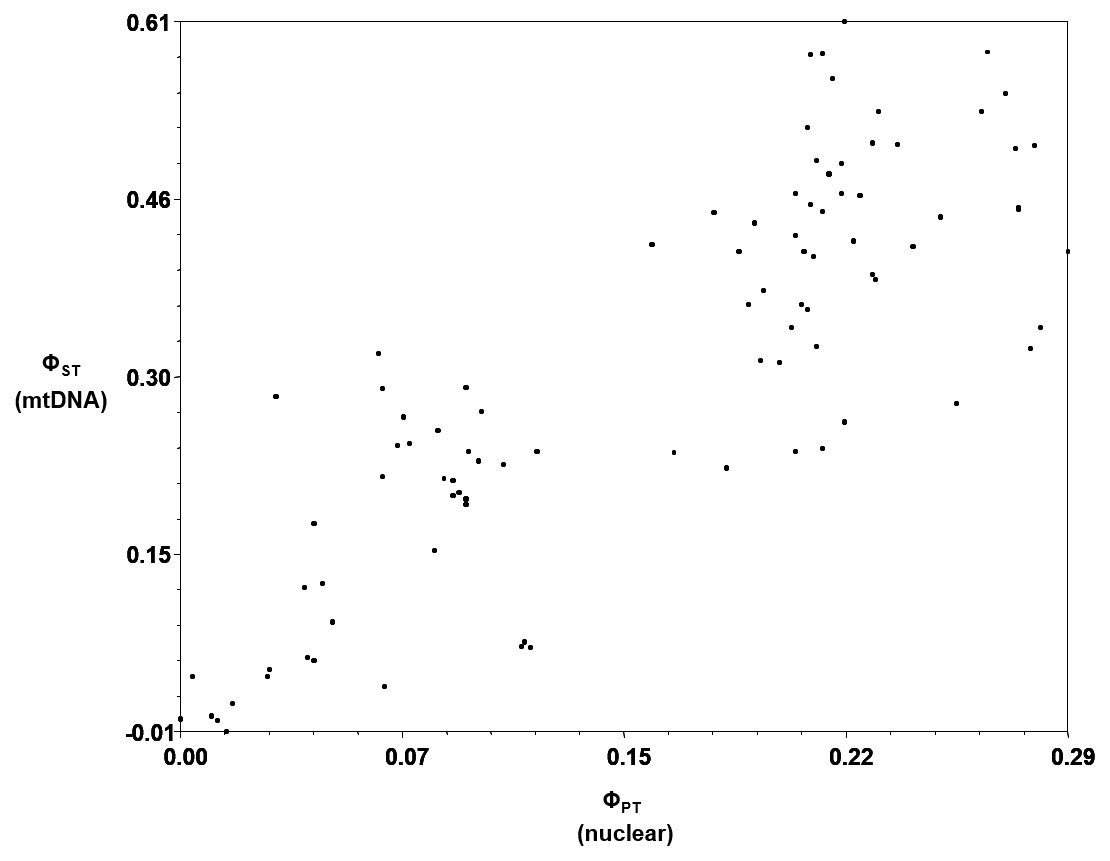

Supplement: Figure S2 — Scatter plot depicting the Mantel matrix regression analysis comparing the mtDNA ΦST matrix for 14 Atlantic coast collections of shortnose sturgeon ( Acipenser brevirostrum ) (Wirgin et al. [30] ) and the nuclear DNA ΦPT pair-wise distance matrix (this study) for the same collections surveyed at 11 polysomic microsatellite DNA loci. The correlation coefficient (r) for this analysis was 0.84 (P<0.0001). (TIF) [file pone.0102784.s002.tif]
