# Supplementary material for: A Nuclear DNA Perspective on Delineating Evolutionarily Significant Lineages in Polyploids: The Case of the Endangered Shortnose Sturgeon (Acipenser brevirostrum)
Source: PLoS One. 2014 Aug 28;9(8):e102784. doi: 10.1371/journal.pone.0102784 (PMC4148239; doi:10.1371/journal.pone.0102784)
Supplement: Table S1 — Pair-wise R values (below diagonal) and Bonferroni-corrected probability values (above diagonal) from the non-parametric Analysis of Similarity (ANOSIM) (Clark 1993) on Jaccard's distance metric (1-Jaccard similarity) measured among 17 collections of shortnose sturgeon ( Acipenser brevirostrum ) surveyed at 11 polysomic microsatellite loci. (DOC) [file pone.0102784.s003.doc]

Table S1. Pair-wise R values (below diagonal) and Bonferroni-corrected probability values (above diagonal) from the non-parametric Analysis of Similarity (ANOSIM) (Clark 1993) on Jaccard’s distance metric (1-Jaccard similarity) measured among 17 collections of shortnose sturgeon (*Acipenser brevirostrum*) surveyed at 11 polysomic microsatellite loci.

| **Collection** | **Saint John** | **Penobscot** | **Androscoggin** | **Kennebec** | **Merrimack** | **Connecticut** | **Hudson** | **Delaware** | **Chesapeake Bay** | **Cape Fear** | **Winyah Bay** | **Santee-Cooper** | **Lake Marion** | **Edisto** | **Savannah** | **Ogeechee** | **Altamaha** |
| --- | --- | --- | --- | --- | --- | --- | --- | --- | --- | --- | --- | --- | --- | --- | --- | --- | --- |
| **Saint John** | - | 0.000 | 0.000 | 0.000 | 0.000 | 0.000 | 0.000 | 0.000 | 0.000 | 0.027 | 0.000 | 0.000 | 0.000 | 0.000 | 0.000 | 0.000 | 0.000 |
| **Penobscot** | 0.241 | - | 1.000 | 1.000 | 1.000 | 0.000 | 0.000 | 0.000 | 0.000 | 0.027 | 0.000 | 0.000 | 0.000 | 0.000 | 0.000 | 0.000 | 0.000 |
| **Androscoggin** | 0.345 | 0.099 | - | 1.000 | 0.000 | 0.000 | 0.000 | 0.000 | 0.000 | 0.626 | 0.000 | 0.000 | 0.000 | 0.000 | 0.000 | 0.000 | 0.000 |
| **Kennebec** | 0.285 | 0.014 | 0.058 | - | 0.000 | 0.000 | 0.000 | 0.000 | 0.000 | 0.313 | 0.000 | 0.000 | 0.000 | 0.000 | 0.000 | 0.000 | 0.000 |
| **Merrimack** | 0.395 | 0.089 | 0.306 | 0.189 | - | 0.000 | 0.000 | 0.000 | 0.000 | 0.095 | 0.000 | 0.000 | 0.000 | 0.000 | 0.000 | 0.000 | 0.000 |
| **Connecticut** | 0.793 | 0.513 | 0.617 | 0.573 | 0.770 | - | 0.000 | 0.000 | 0.000 | 0.000 | 0.000 | 0.000 | 0.000 | 0.000 | 0.000 | 0.000 | 0.000 |
| **Hudson** | 0.698 | 0.465 | 0.491 | 0.376 | 0.548 | 0.373 | - | 0.000 | 0.000 | 0.068 | 0.000 | 0.000 | 0.000 | 0.000 | 0.000 | 0.000 | 0.000 |
| **Delaware** | 0.735 | 0.501 | 0.510 | 0.466 | 0.685 | 0.452 | 0.296 | - | 0.000 | 0.041 | 0.000 | 0.000 | 0.000 | 0.000 | 0.000 | 0.000 | 0.000 |
| **Chesapeake Bay** | 0.659 | 0.467 | 0.488 | 0.426 | 0.619 | 0.557 | 0.352 | 0.089 | - | 0.027 | 0.000 | 0.000 | 0.000 | 0.000 | 0.000 | 0.000 | 0.000 |
| **Cape Fear** | 0.780 | 0.644 | 0.473 | 0.466 | 0.933 | 0.860 | 0.610 | 0.678 | 0.629 | - | 1.000 | 1.000 | 1.000 | 1.000 | 1.000 | 1.000 | 1.000 |
| **Winyah Bay** | 0.926 | 0.797 | 0.856 | 0.811 | 0.924 | 0.873 | 0.742 | 0.768 | 0.792 | 0.000 | - | 0.000 | 1.000 | 0.000 | 0.000 | 0.082 | 0.014 |
| **Santee-Cooper** | 0.948 | 0.775 | 0.888 | 0.833 | 0.972 | 0.898 | 0.763 | 0.795 | 0.841 | 0.461 | 0.153 | - | 0.000 | 0.000 | 0.000 | 0.000 | 0.000 |
| **Lake Marion** | 0.902 | 0.714 | 0.817 | 0.746 | 0.946 | 0.882 | 0.727 | 0.737 | 0.752 | 0.267 | 0.058 | 0.159 | - | 0.000 | 0.000 | 0.000 | 0.000 |
| **Edisto** | 0.945 | 0.734 | 0.834 | 0.774 | 0.952 | 0.910 | 0.733 | 0.762 | 0.798 | 0.265 | 0.140 | 0.208 | 0.342 | - | 1.000 | 1.000 | 0.000 |
| **Savannah** | 0.955 | 0.760 | 0.832 | 0.800 | 0.970 | 0.918 | 0.745 | 0.775 | 0.822 | 0.397 | 0.163 | 0.194 | 0.361 | 0.022 | - | 1.000 | 0.000 |
| **Ogeechee** | 0.949 | 0.749 | 0.854 | 0.799 | 0.967 | 0.923 | 0.762 | 0.783 | 0.810 | 0.320 | 0.080 | 0.192 | 0.345 | 0.037 | 0.027 | - | 0.041 |
| **Altamaha** | 0.945 | 0.753 | 0.857 | 0.803 | 0.966 | 0.923 | 0.749 | 0.782 | 0.803 | 0.238 | 0.092 | 0.293 | 0.321 | 0.100 | 0.232 | 0.073 | - |
