# Supplementary material for: A Nuclear DNA Perspective on Delineating Evolutionarily Significant Lineages in Polyploids: The Case of the Endangered Shortnose Sturgeon (Acipenser brevirostrum)
Source: PLoS One. 2014 Aug 28;9(8):e102784. doi: 10.1371/journal.pone.0102784 (PMC4148239; doi:10.1371/journal.pone.0102784)
Supplement: Table S2 — Hierarchical structuring of genetic variation was measured for numerous combinations of shortnose sturgeon ( Acipenser brevirostrum ) collections using analysis of molecular variance (AMOVA). Significance levels of the variance components were based on 1000 permutations. Abbreviations are as follows: NE = Northeast regional grouping includes Saint John River (SJ), Canada, Penobscot, Kennebec, Androscoggin and Merrimack rivers; Mid-Atlantic regional grouping includes the Connecticut (CT), Hudson (H), and Delaware (DE) rivers, and the Chesapeake Bay proper (CB); and the 3) SE = Southeast regional grouping includes the Cape Fear River (CF), Winyah Bay (WB), Santee-Cooper (S-C), Edisto (E), Savannah (S), Ogeechee (O), and Altamaha (ALT) rivers, and Lake Marion (LM). (DOC) [file pone.0102784.s004.doc]

Table S2. Hierarchical structuring of genetic variation was measured for numerous combinations of shortnose sturgeon (*Acipenser brevirostrum*) collections using analysis of molecular variance (AMOVA). Significance levels of the variance components were based on 1000 permutations. Abbreviations are as follows: NE = Northeast regional grouping includes Saint John River (SJ), Canada, Penobscot, Kennebec, Androscoggin and Merrimack rivers; Mid-Atlantic regional grouping includes the Connecticut (CT), Hudson (H), and Delaware (DE) rivers, and the Chesapeake Bay proper (CB); and the 3) SE = Southeast regional grouping includes the Cape Fear River (CF), Winyah Bay (WB), Santee-Cooper (S-C), Edisto (E), Savannah (S), Ogeechee (O), and Altamaha (ALT) rivers, and Lake Marion (LM).

| **Model** | **Source of Variance** | **Percentage of**  **Variance** | **Test**  **Statistic** | **Value** | **Probability** |
| --- | --- | --- | --- | --- | --- |
| 17 individual populations | Among pops within groupings | 16% | Φ_PT_ | 0.164 | 0.001 |
|  | Within pops | 84% |  |  |  |
|  |  |  |  |  |  |
| 1) 17 populations as 3 groupings | Among groupings | 16% | Φ_RT_ | 0.158 | 0.001 |
| (NE; Mid-Atlantic; SE) | Among pops within groupings | 5% | Φ_PR_ | 0.057 | 0.001 |
|  | Within pops | 79% | Φ_PT_ | 0.206 | 0.001 |
|  |  |  |  |  |  |
| 2) 16 populations as 3 groupings | Among groupings | 16% | Φ_RT_ | 0.158 | 0.001 |
| (#2 with SJ omitted) | Among pops within groupings | 4% | Φ_PR_ | 0.054 | 0.001 |
|  | Within pops | 80% | Φ_PT_ | 0.203 | 0.001 |
|  |  |  |  |  |  |
| 3) 17 populations as 5 groupings | Among groupings | 16% | Φ_RT_ | 0.164 | 0.001 |
| (NE, CT, Hudson, DE/CB, SE) | Among pops within groupings | 4% | Φ_PR_ | 0.042 | 0.001 |
|  | Within pops | 80% | Φ_PT_ | 0.199 | 0.001 |
|  |  |  |  |  |  |
| 4) 16 populations as 5 groupings | Among groupings | 17% | Φ_RT_ | 0.166 | 0.001 |
| (#3 with SJ omitted) | Among pops within groupings | 3% | Φ_PR_ | 0.037 | 0.001 |
|  | Within pops | 80% | Φ_PT_ | 0.196 | 0.001 |
|  |  |  |  |  |  |
| 5) 17 populations as 6 groupings | Among groupings | 17% | Φ_RT_ | 0.167 | 0.001 |
| (#4 with SJ as a grouping) | Among pops within groupings | 3% | Φ_PR_ | 0.036 | 0.001 |
|  | Within pops | 80% | Φ_PT_ | 0.197 | 0.001 |
|  |  |  |  |  |  |
| 6) 16 populations as 6 groupings | Among groupings | 17% | Φ_RT_ | 0.169 | 0.001 |
| (#4 omits SJ and has Merrimack as a grouping) | Among pops within groupings | 3% | Φ_PR_ | 0.031 | 0.001 |
|  | Within pops | 80% | Φ_PT_ | 0.195 | 0.001 |
|  |  |  |  |  |  |
| 7) 17 populations as 7 groupings | Among groupings | 17% | Φ_RT_ | 0.170 | 0.001 |
| (#3 with SJ and Merrimack as groupings) | Among pops within groupings | 3% | Φ_PR_ | 0.031 | 0.001 |
|  | Within pops | 80% | Φ_PT_ | 0.196 | 0.001 |
|  |  |  |  |  |  |
| 8) 17 populations as 6 groupings | Among groupings | 15% | Φ_RT_ | 0.154 | 0.001 |
| (#3 with Altamaha as grouping) | Among pops within groupings | 4% | Φ_PR_ | 0.042 | 0.001 |
|  | Within pops | 81% | Φ_PT_ | 0.190 | 0.001 |
|  |  |  |  |  |  |
| 9) 17 populations as 8 groupings | Among groupings | 15% | Φ_RT_ | 0.145 | 0.001 |
| (NE; CT ; Hudson ; DE/CB ; | Among pops within groupings | 3% | Φ_PR_ | 0.034 | 0.001 |
| CF/WB ; S-C/LM; E-S-O; ALT) | Within pops | 83% | Φ_PT_ | 0.175 | 0.001 |
|  |  |  |  |  |  |
| 10) 17 populations as 9 groupings | Among groupings | 15% | Φ_RT_ | 0.152 | 0.001 |
| (#9 with SJ as a grouping) | Among pops within groupings | 2% | Φ_PR_ | 0.025 | 0.001 |
|  | Within pops | 83% | Φ_PT_ | 0.173 | 0.001 |
|  |  |  |  |  |  |
| 11) 16 populations as 8 groupings | Among groupings | 15% | Φ_RT_ | 0.148 | 0.001 |
| (#10 with SJ omitted) | Among pops within groupings | 2% | Φ_PR_ | 0.025 | 0.001 |
|  | Within pops | 83% | Φ_PT_ | 0.169 | 0.001 |
