# Supplementary material for: A Nuclear DNA Perspective on Delineating Evolutionarily Significant Lineages in Polyploids: The Case of the Endangered Shortnose Sturgeon (Acipenser brevirostrum)
Source: PLoS One. 2014 Aug 28;9(8):e102784. doi: 10.1371/journal.pone.0102784 (PMC4148239; doi:10.1371/journal.pone.0102784)
Supplement: Table S3 — Assignment to collection of origin for 17 shortnose sturgeon ( Acipenser brevirostrum ) collections surveyed at 11 polysomic microsatellite DNA markers. Mis-assigned individuals are distributed horizontally. (DOC) [file pone.0102784.s005.doc]

Table S3. Assignment to collection of origin for 17 shortnose sturgeon (*Acipenser brevirostrum*) collections surveyed at 11 polysomic microsatellite DNA markers. Mis-assigned individuals are distributed horizontally.

| **Allocated to** | **Saint John** | **Penobscot** | **Androscoggin** | **Kennebec** | **Merrimack** | **Connecticut** | **Hudson** | **Delaware** | **Chesapeake Bay** | **Cape Fear** | **Winyah Bay** | **Santee-Cooper** | **Lake Marion** | **Edisto** | **Savannah** | **Ogeechee** | **Altamaha** |
| --- | --- | --- | --- | --- | --- | --- | --- | --- | --- | --- | --- | --- | --- | --- | --- | --- | --- |
| **Saint John** | 20 | 1 | 0 | 0 | 0 | 0 | 0 | 0 | 0 | 0 | 0 | 0 | 0 | 0 | 0 | 0 | 0 |
| **Penobscot** | 2 | 27 | 9 | 10 | 3 | 0 | 0 | 0 | 0 | 0 | 0 | 0 | 0 | 0 | 0 | 0 | 0 |
| **Androscoggin** | 1 | 5 | 10 | 2 | 0 | 0 | 0 | 0 | 0 | 0 | 0 | 0 | 0 | 0 | 0 | 0 | 0 |
| **Kennebec** | 2 | 6 | 4 | 11 | 2 | 0 | 0 | 0 | 0 | 0 | 0 | 0 | 0 | 0 | 0 | 0 | 0 |
| **Merrimack** | 0 | 0 | 0 | 0 | 17 | 0 | 0 | 0 | 0 | 0 | 0 | 0 | 0 | 0 | 0 | 0 | 0 |
| **Connecticut** | 0 | 0 | 0 | 0 | 0 | 46 | 0 | 0 | 0 | 0 | 0 | 0 | 0 | 0 | 0 | 0 | 0 |
| **Hudson** | 0 | 0 | 0 | 0 | 0 | 1 | 44 | 2 | 3 | 0 | 0 | 0 | 0 | 0 | 0 | 0 | 0 |
| **Delaware** | 0 | 0 | 0 | 0 | 0 | 0 | 1 | 29 | 8 | 0 | 0 | 0 | 0 | 0 | 0 | 0 | 0 |
| **Chesapeake Bay** | 0 | 0 | 0 | 1 | 0 | 0 | 0 | 8 | 23 | 0 | 0 | 0 | 0 | 0 | 0 | 0 | 0 |
| **Cape Fear** | 0 | 0 | 0 | 0 | 0 | 0 | 0 | 0 | 0 | 0 | 0 | 0 | 0 | 0 | 0 | 0 | 0 |
| **Winyah Bay** | 0 | 0 | 0 | 0 | 0 | 0 | 0 | 0 | 0 | 3 | 29 | 3 | 5 | 2 | 0 | 2 | 2 |
| **Santee-Cooper** | 0 | 0 | 0 | 0 | 0 | 0 | 0 | 0 | 0 | 0 | 3 | 24 | 4 | 1 | 2 | 2 | 2 |
| **Lake Marion** | 0 | 0 | 0 | 0 | 0 | 0 | 0 | 0 | 0 | 0 | 4 | 3 | 22 | 1 | 1 | 0 | 0 |
| **Edisto** | 0 | 0 | 0 | 0 | 0 | 0 | 0 | 0 | 0 | 0 | 3 | 4 | 0 | 13 | 8 | 6 | 4 |
| **Savannah** | 0 | 0 | 0 | 0 | 0 | 0 | 0 | 0 | 0 | 0 | 3 | 4 | 0 | 3 | 12 | 7 | 0 |
| **Ogeechee** | 0 | 0 | 0 | 0 | 0 | 0 | 0 | 0 | 0 | 0 | 1 | 3 | 0 | 7 | 9 | 10 | 9 |
| **Altamaha** | 0 | 0 | 0 | 0 | 0 | 0 | 0 | 0 | 0 | 0 | 4 | 1 | 2 | 6 | 2 | 8 | 19 |
| **Correct**  **Assignment %** | 80.0 | 69.2 | 43.5 | 45.8 | 77.3 | 97.9 | 97.8 | 74.4 | 67.6 | 0.0 | 61.7 | 58.5 | 66.7 | 39.4 | 35.3 | 28.6 | 52.8 |
| **Overall assignment to collection** 58.6% | | | | | | | | | | | | | | | | | |
