# Supplementary material for: A Nuclear DNA Perspective on Delineating Evolutionarily Significant Lineages in Polyploids: The Case of the Endangered Shortnose Sturgeon (Acipenser brevirostrum)
Source: PLoS One. 2014 Aug 28;9(8):e102784. doi: 10.1371/journal.pone.0102784 (PMC4148239; doi:10.1371/journal.pone.0102784)
Supplement: Table S4 — Assignment to proposed grouping (five groupings model) in shortnose sturgeon ( Acipenser brevirostrum ) surveyed at 11 polysomic microsatellite DNA markers. The overall correct assignment rate to proposed grouping was 99.1% (522/527). Mis-assigned individuals are distributed vertically. Northeast regional grouping includes Saint John River (SJ), Canada, Penobscot, Kennebec, Androscoggin and Merrimack rivers; and the Southeast regional grouping includes the Cape Fear River (CF), Winyah Bay (WB), Santee-Cooper (S-C), Edisto (E), Savannah (S), Ogeechee (O), and Altamaha (ALT) rivers, and Lake Marion (LM). (DOC) [file pone.0102784.s006.doc]

Table S4. Assignment to proposed grouping (five groupings model) in shortnose sturgeon (*Acipenser brevirostrum*) surveyed at 11 polysomic microsatellite DNA markers. The overall correct assignment rate to proposed grouping was 99.1% (522/527). Mis-assigned individuals are distributed vertically. Northeast regional grouping includes Saint John River (SJ), Canada, Penobscot, Kennebec, Androscoggin and Merrimack rivers; and the Southeast regional grouping includes the Cape Fear River (CF), Winyah Bay (WB), Santee-Cooper (S-C), Edisto (E), Savannah (S), Ogeechee (O), and Altamaha (ALT) rivers, and Lake Marion (LM).

| **Allocated to** | **Northeast** | **Connecticut** | **Hudson** | **Delaware** | **Southeast** |
| --- | --- | --- | --- | --- | --- |
| **Northeast** | 131 | 0 | 0 | 0 | 0 |
| **Connecticut** | 0 | 46 | 0 | 0 | 0 |
| **Hudson** | 0 | 1 | 44 | 2 | 0 |
| **Delaware** | 1 | 0 | 1 | 71 | 0 |
| **Southeast** | 0 | 0 | 0 | 0 | 254 |
| **Assignment %** | 99.1 | 97.9 | 97.8 | 97.3 | 100.0 |
